# Catastrophizing Thoughts and Fear-Avoidance Behavior Are Related to Persistent Post-Concussion Symptoms after Mild Traumatic Brain Injury

**DOI:** 10.1089/neur.2024.0136

**Published:** 2025-02-06

**Authors:** Lynn Hecker, Skye King, Melloney Wijenberg, Chantal Geusgens, Sven Stapert, Jeanine Verbunt, Caroline Van Heugten

**Affiliations:** ^1^ Department of Clinical and Medical Psychology, Zuyderland Medical Centre, Sittard-Geleen, the Netherlands.; ^2^ Department of Neuropsychology and Psychopharmacology, Faculty of Psychology and Neuroscience, Maastricht University, Maastricht, the Netherlands.; ^3^ Limburg Brain Injury Centre, Maastricht University, Maastricht, the Netherlands.; ^4^ Adelante, Center of Expertise in Rehabilitation and Audiology, Hoensbroek, the Netherlands.; ^5^ Department of Rehabilitation Medicine, Care and Public Health Research Institute (CAPHRI), Maastricht University, Maastricht, the Netherlands.

**Keywords:** catastrophizing, fear, mild traumatic brain injury, post-concussion symptoms

## Abstract

A small percentage of patients with mild traumatic brain injury (mTBI) does not follow the expected recovery trajectory but develop persistent post-concussion symptoms (PCS). The fear-avoidance model (FAM) is a general biopsychosocial model that may potentially explain the development and continuation of persistent PCS for a subgroup of patients. The aim of the present study was to investigate if the FAM can (at least partially) explain PCS at 3 and 6 months post-mTBI by investigating associations between the elements of the FAM. A prospective, longitudinal, multicenter cohort study with outcome assessments at 2 weeks, 3 months, and 6 months post-mTBI was conducted in 163 patients with mTBI recruited from the emergency department and neurology department within 2 weeks post-mTBI. The FAM components PCS, catastrophizing, fear-avoidance behavior and depressive symptoms correlated significantly with each other at 3 months post-mTBI (*p* < 0.01) and correlations ranged from 0.40 to 0.72. No significant correlations were found between disuse and the other components. Depressive symptoms at 3 months post-mTBI significantly correlated with PCS at 6 months post-mTBI. Our results suggest that the FAM could be an explanatory model for the development of persistent PCS. This implies that treatment development for patients with persistent PCS could be aimed at the components of the FAM, such as exposure therapy to reduce catastrophizing and avoidance behavior.

## Introduction

The vast majority of patients with mild traumatic brain injury (mTBI) recover quickly in the first few weeks to months without any lasting symptoms 3 months later.^1–3^ However, a minority of mTBI patients report a delay in recovery, persistent symptoms, and impaired functioning in daily life for months, if not years, after mTBI.^4^ The prevalence of this group varies between 15% and 47% in different studies depending on the study methodology and due to inconsistencies regarding its definition.^5–7^ Since the incidence of mTBI is high, this percentage still results in a group of patients who are on sick leave from work and often seek help within health care, which leads to high societal costs.^8^ These patients suffer from chronic cognitive, emotional, and somatic symptoms such as attention and memory weakness, irritability, sensitivity to noise, anxiety, mood instability, depression, fatigue, dizziness, sleep impairment, and headache.^9^

Persistent symptoms after mTBI are controversial in health care as there is no clear understanding of the origin, evolution, duration, or resolution of these symptoms.^10^ Psychological variables, social factors, and psychiatric vulnerabilities appear to be stronger and more consistent predictors for the development of persistent symptoms than biological factors alone.^11–13^ Pre-injury mental health status, anxiety levels, early post-injury stress, and maladaptive coping styles such as fear-avoidance behavior (i.e., the avoidance of physical or mental activities based on the anticipated fear of increased symptoms) have been associated with poorer outcome after mTBI.^11,14–16^

Furthermore, several studies have shown that the symptoms related to mTBI are not specific for brain injury as they are also prevalent in orthopedic injuries,^17^ chronic pain,^18^ and whiplash injuries.^19^ The symptoms are even known to occur in the healthy population in the absence of disease.^20^ Thus, these persistent symptoms are not mTBI specific and therefore they are unlikely to be explained by biomedical factors alone (i.e., the mTBI). Therefore, biopsychosocial models should be considered in understanding the development and nature of persistent symptoms after mTBI.

**FIG. 1.**
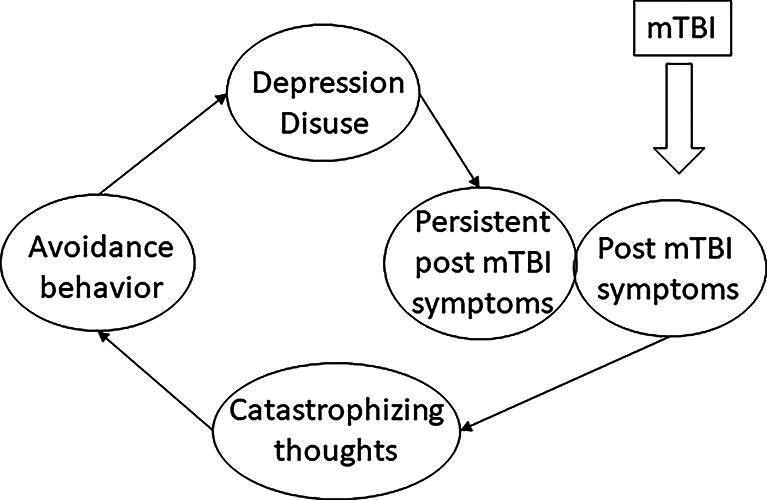
Proposed fear-avoidance model for persistent symptoms after mTBI. mTBI, mild traumatic brain injury.

In several other bodily distress syndromes, including chronic pain,^21^ tinnitus,^22,23^ chronic fatigue,^24^ fibromyalgia,^24^ and fatigue in multiple sclerosis^25,26^ the biopsychosocial fear-avoidance model (FAM) contributes to explaining the chronic symptoms. This model shows how fear is the anticipatory emotional response to threat, and how adaptive learning takes place through either experience, observation, or information. Persistent symptoms may develop when symptom-related fear and avoidance persist alongside biological recovery, or when protective responses generalize to novel situations and become routine behavior.^27^ In mTBI, a cognitive fear-avoidance cycle tends to occur when the cognitive symptoms are erroneously interpreted as evidence of pathology over which one has little or no control.^28,29^ This catastrophizing could extend to fear and avoidance of mental activities, also known as cogniphobia,^30^ which subsequently leads to a decrease of activity levels as a result of avoidance and may lead to disuse, isolation, disability, and depression. Consequently, attention for the symptoms can increase over time and this results in worsening of catastrophizing and avoidance behavior, creating a vicious cycle (see Fig. 1).

The FAM has already been proposed as a possible explanation for persistent symptoms after TBI.^15,31^ The study of Wijenberg and colleagues^15^ used a cross-sectional, retrospective design to show that post-concussion symptoms (PCS), catastrophizing thoughts, fear-avoidance behavior and depressive symptoms correlated significantly in patients with TBI. The correlations were even stronger for mTBI than for moderate-to-severe TBI. Another study on the applicability of the FAM in mTBI by Silverberg and colleagues^31^ found that patients who engage in fear-avoidance behavior in the weeks to months after mTBI appear to have an increased risk for developing persistent symptoms, disability, and adverse mental health outcomes such as depression and anxiety. Both studies have some limitations that will be addressed in this study. One limitation is that they demonstrated the FAM in patient populations that consulted medical care for their persistent symptoms after TBI. Patients seen in these settings are more likely to have adverse psychological risk profiles in comparison with patients recruited from the emergency department for purposes of follow-up recovery.^32^ Furthermore, both Wijenberg^15^ and Silverberg^31^ included participants at variable time points after TBI leading to a heterogeneous group of participants in different stages of their recovery or disease process. If patients still have symptoms at 3 months post-mTBI and onwards, the symptoms are by consensus classified as persistent.^1–3^

Furthermore, both studies^15,31^ used a cross-sectional design. As a consequence, the effect of the cycle, i.e., further increase/persistence of symptoms over time, could not be taken into account but only speculated upon. Therefore, we add to existing knowledge by using a longitudinal design in the current study.

To further test the assumptions of the FAM in explaining persistent symptoms after mTBI, we replicate the earlier correlational studies in a prospectively collected mTBI sample. The current study focused on the frequency and relationships of PCS after mTBI and the main elements of the FAM model, i.e., catastrophizing thoughts, fear-avoidance behavior, depression symptoms, and disuse, at 3 months after mTBI. We studied this in mTBI patients recruited at the emergency or neurology department within 14 days after the injury was sustained, irrespective of their recovery trajectory. To investigate the increase of symptoms over time as expected in the vicious cycle of the FAM, a follow-up of the post-mTBI symptoms at 6 months was taken into account.

The aim of this study was two-fold: (1) to investigate the cross-sectional relationship between all components of the FAM (i.e., PCS, catastrophizing thoughts, fear-avoidance behavior, depression, and disuse) at 3 months post-mTBI and (2) to investigate the longitudinal relationships between both depression and disuse of the FAM at 3 months post-mTBI and the PCS at 6 months post-mTBI.

We hypothesized that there would be a significant and positive correlation between the components incorporated in the FAM at 3 months post-mTBI: PCS, catastrophizing thoughts, fear-avoidance behavior, depression, and disuse. We further hypothesized that there would be a significant and positive correlation between both depression and disuse of the FAM at 3 months post-mTBI and PCS 6 months post-mTBI (see Fig. 1).

## Materials and Methods

In this prospective, longitudinal, multicenter, cohort study on long-term consequences of mTBI, data were collected at time points T1 (within 2 weeks post-mTBI), T2 (3 months post-mTBI), T3 (6 months post-mTBI) and T4 (12 months post-mTBI). For the current study, the data from, T1, T2, and T3 were included in the analysis. Ethical approval was received from the medical ethics committee of Maastricht University and Maastricht University Medical Centre (METC 16-4-209) and the participating centers.

### Participants

Participants with an mTBI who visited the emergency department or neurology department at one of the six participating hospitals (“Laurentius Ziekenhuis Roermond,” “Maastricht UMC+,” “St Jans Gasthuis Weert,” “VieCurie Medisch Centrum Venlo,” “Zuyderland Heerlen,” and “Zuyderland Sittard”) between March 2017 and August 2019 were eligible to participate in the cohort study. The following inclusion criteria were used: (1) diagnosed with mTBI by their treating health care professional using the World Health Organization and European Federation of Neurological Societies criteria at the neurology or emergency department^32,33^ that include: history of impact to the head, Glasgow Coma Scale (GCS) score between 13 and 15, 30 min after the impact or later at hospital admission and in case of a GCS score of 15, at least one of the following: loss of consciousness (LOC) (≤30 min), post-traumatic amnesia (PTA) (≤24 h), and other transient neurological signs such as vomiting; (2) able and willing to provide informed consent; (3) fluent in Dutch; and (4) aged 18 years or older.

Exclusion criteria were as follows: (1) a history of neurological disease or injury such as epilepsy and multiple sclerosis; (2) a history of psychiatric disorders for which hospitalization was needed; (3) under the influence of illicit substances at the time of injury or a history of drug addiction; and (4) use of psychoactive medication known for cognitive (side) effects.

### Procedure

Potential participants were given information about the study by their treating physician following diagnosis of mTBI at the emergency or neurology department of the participating hospital. After a patient expressed interest in the study, contact details were forwarded to the research team who scheduled a first measurement within 2 weeks after the injury (T1). This took place at the participant’s home or at the university. At this point, the inclusion and exclusion criteria were checked and the informed consent was signed, where after the questionnaires were completed in an online testing environment. The researcher could provide help to navigate this online system. After completing the first measurement, participants received a notification for the upcoming follow-ups: after 3 months (T2) and 6 months (T3). Participants received an email with a link to the online questionnaires which they completed themselves. Participants were given 4 weeks to complete the questionnaires after each time point. If the questionnaires had not been completed within 2 weeks after receiving them, the participant received a reminder email or phone call. If help was needed, the researcher could be contacted and assistance at home was available.

### Measures

#### Basic demographic information

Several demographics (age, gender, education, and psychological treatment history) were reported by the participants at T1, and injury-related variables (injury severity parameters, cause of injury, or intracerebral abnormality on computed tomography [CT] or magnetic resonance imaging [MRI]) were retrieved from the hospital database. Education was classified using the Dutch classification system.^34^

#### PCS assessed at 3 (T2) and 6 (T3) months

PCS were assessed using the Dutch version of the Rivermead Post-Concussion Symptoms Questionnaire (RPQ).^35^ The RPQ assesses the frequency and severity of PCS. It is commonly used to evaluate the severity of remaining symptoms after an mTBI, and it is a valid and reliable measure.^36^ Patients are asked to compare the severity of currently experienced PCS with premorbid levels. The RPQ includes 16 items that are rated on a 4-point Likert Scale ranging from “not experienced at all” (0) to a “severe problem” (4). The total score ranges from 0 from 64. PCS was defined as the presence of three or more residual symptoms, as evidenced by an item score of two or higher. Previous studies have utilized the same criterion.^37–41^

The following elements of the FAM model were assessed at 3 months (T2).

#### Catastrophizing thoughts

Catastrophizing about PCS was assessed using the Post-Concussion Symptoms Catastrophizing Scale (PCS-CS) which has adequate psychometrics properties.^42^ This is an adaption of the Dutch translation of the Pain Catastrophizing Scale.^43,44^ The PCS was adapted by replacing the word “pain” with common PCS in all items: “headaches, dizziness, fatigue, memory and concentration problems.” The PCS-CS consists of 13 items that use a 5-point Likert scale to assess the self-reported frequency of catastrophizing thoughts about mTBI symptoms. The scale ranges from 0 to 52, with higher scores indicating more intense catastrophizing.

#### Fear-avoidance behavior

Concussion-related fear-avoidance behavior was assessed with the Fear of Mental Activity scale (FMA), which has adequate psychometric properties.^42^ The FMA is developed based on the valid and reliable Dutch version of the Tampa Scale for Kinesiophobia (TSK).^45–47^ The TSK was modified by replacing the word “pain” in all items with the following common PCS: “headaches, dizziness, fatigue, memory and concentration problems.” Additionally, items were changed to be more appropriate for mTBI, such as “My head tells me there is something dangerously wrong,” rather than “My body tells me there is something dangerously wrong.” The questionnaire consists of 17 items, scored on a 4-point scale with a total score ranging from 17 to 68.

#### Depressive symptoms

Depressive symptoms were assessed using the subscale depression of the Dutch version of the Hospital Anxiety and Depression Scale (HADS).^48^ It is a valid and reliable measure for screening depression in patients with mTBI.^49^ The depression subscale consists of seven items that are scored on a 4-point Likert scale, yielding a score range of 0 to 21. A higher score indicates a higher intensity of depression. A score of 8 or higher is an indication for depression in the normal population as well as in patients with TBI.^49^

#### Disuse

Validated questionnaires to assess “disuse of the brain” are lacking. Therefore, mTBI-related disuse was operationalized as “the number of hours of mental activity per day.” Disuse was assessed on T1 using the question “How much time did you spent on mental activities per day before the accident?” On T2 and T3, the question was “How much time do you spent on mental activities per day?” In this study, exposure to mental activity represented the inverse of “disuse” indicated by the number of hours spent on mental activity per day. In other words, the scores were reversed to represent disuse. Disuse was calculated using the score on T1 minus the score on T2 (hours of mental activity per day on T1—hours of mental activity per day on T2). There is no agreed-upon threshold for “disuse.”^50^

### Statistical analyses

Data analyses were performed using IBM SPSS IBM SPSS version 28.0. The data had a normal distribution, and there were no restrictions of range. Demographic and injury-related characteristics are presented using descriptive statistics. The mean and standard deviation of PCS (RPQ) (T2 and T3), depression (HADS-D) (T2), disuse (number of hours) (T2), catastrophizing (PCS-CS) (T2) and fear-avoidance behavior (FMA) (T2), and the percentage of participants with PCS (T2 and T3) are presented using descriptive statistics. Pearson correlation coefficients were calculated to show relationships between the components of the FAM at T2: RPQ, PCS-CS, FMA, HADS-D, and the disuse question. In addition, Pearson correlation coefficients were calculated between both disuse and depression of the FAM at T2 (HADS-D, disuse question) and the PCS at T3 (RPQ) to investigate the vicious cycle of the FAM over time. Correlation coefficients are defined as small from 0.10 to 0.29, moderate from 0.30 to 0.49, and large from 0.50 (Cohen, 1988). For all statistical tests, an alpha level of 0.05 was used.

## Results

### Patient sample

**FIG. 2.**
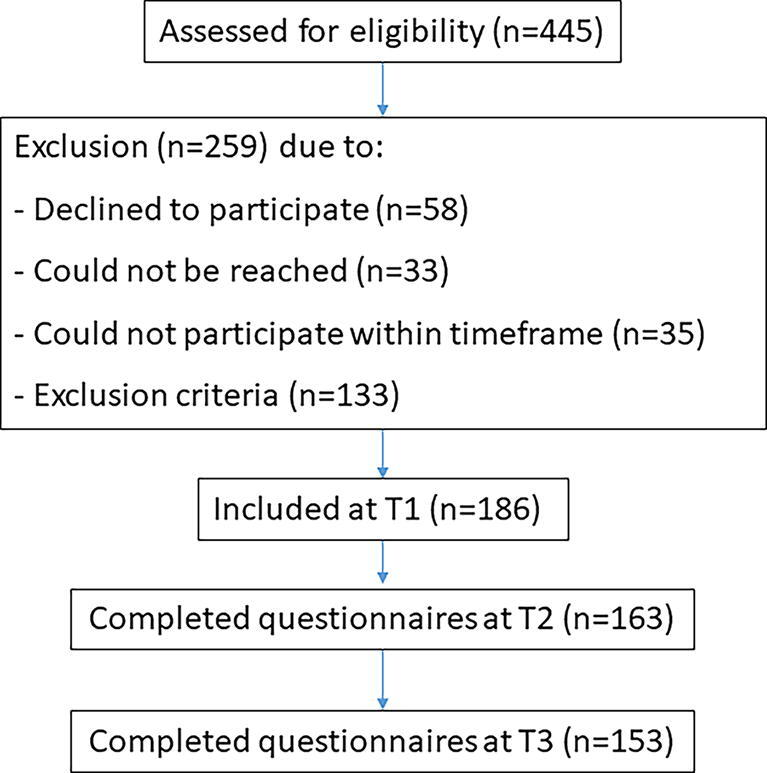
Participant flowchart.

A total of 445 patients with mTBI were assessed for eligibility, 133 patients did not meet the inclusion criteria and 186 patients met the inclusion criteria and were willing to participate. At 3 months post-injury (T2), 163 patients with mTBI completed the questionnaires, which is the sample of participants entered into the analyses for this study. See Figure 2 for a participant flowchart.

Table 1 shows the general and injury-related characteristics of the sample that participated at T2.

**Table 1.** General and Injury-Related Characteristics (*n* = 163)

Variables	*N* (%) or mean (SD)
Gender (males)	100 (61.3)
Age, mean (SD) in years	50.4 (26.4)
High education	61 (37.4)
Psychiatric treatment	
Current (yes)	6 (3.7)
Past (yes)	21 (12.9)
Cause of injury	
Fall	63 (38.7)
Traffic	70 (42.9)
Violence	7 (4.3)
Sport	18 (11)
Other	5 (3.0)
GCS (13–15)	14.9 (0.4)
LOC in minutes	
*N* (%)	99 (60.7)
Duration (mean, SD) (*n* = 142)	3.3 (5.2)
PTA in hours	
*N* (%)	104 (63.8)
Duration (mean, SD) (*n* = 154)	1.9 (4.3)
CT abnormalities (*n*, %)	19 (11.7)

CT, computed tomography, observing potential damage; GCS, Glasgow Coma Scale; LOC, loss of consciousness; PTA, post-traumatic amnesia; SD, standard deviation.

In most cases, mTBI was caused by traffic accidents (42.5%). The majority (60.8%) of patients with mTBI experienced LOC, with a mean duration of 3.3 min. Conform the criteria of mTBI, the majority of participants (64.5%) experienced PTA, with a mean duration of 1.9 h (SD = 4.3).

### Frequency of PCS, catastrophizing, fear-avoidance behavior, depression, and disuse

Table 2 shows the prevalence of PCS on T2 and T3. Fatigue, reduced ability to concentrate, forgetfulness, sleep disturbances, and headaches are the five most reported symptoms at 3 months post-mTBI. At 6 months post-mTBI, the five most reported symptoms remained the same, except for the disappearance of headaches and the addition of slowing of thought processes.

**Table 2.** Prevalence of Post-Concussion Symptoms at 3 and 6 Months Post-mTBI

Post-concussion symptoms (RPQ)	T2 (*n* = 163) *n* (%)	T3 (*n* = 153) *n* (%)
Headaches (RPQ1)^a^	49 (30)	37 (24.2)
Dizziness (RPQ2)	41 (25.2)	28 (18.3)
Nausea (RPQ3)	10 (6.1)	6 (3.9)
Increased sensitivity to noise (RPQ4)	28 (17.2)	21 (13.7)
Sleep disturbances (RPQ5)^a,b^	49 (30.1)	46 (30.1)
Fatigue (RPQ6)^a,b^	70 (43)	55 (35.9)
Irritability (RPQ7)	31 (19)	32 (20.9)
Feeling depressed/teary-eyed (RPQ8)	27 (16.6)	29 (19.0)
Feeling impatient or frustrated (RPQ9)	32 (19.6)	32 (20.9)
Forgetfulness (RPQ10)^a,b^	52 (31.9)	49 (32.0)
Reduced ability to concentrate (RPQ11)^a,b^	55 (33.7)	46 (30.1)
Slowing of thought and processes (RPQ12)^b^	44 (27)	40 (26.1)
Blurred vision (RPQ13)	19 (11.7)	24 (15.7)
Increased sensitivity to light (RPQ14)	17 (10.4)	14 (9.2)
Double vision (RPQ15)	7 (4.3)	8 (5.2)
Feeling agitated/restless (RPQ16)	27 (16.6)	27 (17.6)

Symptom with a score of 2 or higher was included.

^a^ Top five ranking of most reported symptoms at T2.

^b^ Top five ranking of the most reported symptoms at T3.

mTBI, mild traumatic brain injury; RPQ, Rivermead Post-Concussion Symptoms Questionnaire.

Table 3 shows the scores on the PCS-CS, FMA, HADS depression subscale, the disuse question on T2, and the RPQ on T2 and T3. A total of 44% of patients fulfilled the criterion of PCS (having three or more PCS) on T2 and this decreased to 38% on T3. A total of 33% fulfilled the criterion of PCS on both T2 and T3. A total of 8% of the participants reported depressive symptoms at a clinically significant level (total score HADS depression 8 or higher).

**Table 3.** The Descriptive Statistics of All Variables of the Fear-Avoidance Model

	mTBI (*n* T2 = 163, *n* T3 = 153)		
Variables	Mean	SD	Range
Post-concussion symptoms 3 months post-mTBI (RPQ T2)	10.4	11.2	0–46
Catastrophizing (PCS-CS T2)	5.4	8.4	0–52
Fear avoidance (FMA T2)	16.4	5.4	13–40
Depression (HADS-D T2)	2.5	3.3	0–16
Disuse question (T1 − T2)	−0.7	3	−9 to 7
Post-concussion symptoms 6 months post-mTBI (RPQ T3)	9.8	11.2	0–50

FMA, Fear of Mental Activity scale; HADS-D, Depression subscale of the Hospital Anxiety and Depression Scale; mTBI, mild traumatic brain injury; PCS-CS, Post-Concussion Symptoms Catastrophizing Scale; RPQ, Rivermead Post-Concussion Symptoms Questionnaire.

### Relationships between the components in the FA model

At T2, all components of the FAM significantly correlated with each other (*p* < 0.01), except for disuse which did not correlate with any of the other components (see Table 4). There is a strong correlation between the component depression of the FAM at T2 and the PCS at T3. The strongest association was seen between PCS on T2 and catastrophizing at T2 (*r =* 0.72).

**Table 4.** Correlations of All Components of the Fear-Avoidance Model

	mTBI (*n* T2 = 163, *n* T3 = 153)					
Variables	RPQ T2	PCS-CS T2	FMA T2	HADS-D T2	Disuse question T1 − T2	RPQ T3
Post-concussion symptoms 3 months post-mTBI (RPQ T2)	—	0.72^*^	0.60^*^	0.68^*^	0.01	0.85^*^
Catastrophizing (PCS-CS T2)	—	—	0.69^*^	0.65^*^	−0.002	—
Fear avoidance (FMA T2)	—	—	—	0.51^*^	0.07	—
Depression (HADS-D T2)	—	—	—	—	0.001	0.63^*^
Disuse question (T1 − T2)	—	—	—	—	—	−0.03
Post-concussion symptoms 6 months post-mTBI (RPQ T3)	—	—	—	—	—	—

^*^ *p* < 0.01.

FMA, Fear of Mental Activity scale; HADS-D, Depression subscale of the Hospital Anxiety and Depression Scale; mTBI, mild traumatic brain injury; PCS-CS, Post-Concussion Symptoms Catastrophizing Scale; RPQ, Rivermead Post-Concussion Symptoms Questionnaire.

## Discussion

In this study, we investigated the assumption that the FAM may—at least partially—explain persistent symptoms in patients with mTBI. The results showed a significant and positive correlation between PCS, catastrophizing thoughts, fear-avoidance behavior, and depression at 3 months post-mTBI. This is in line with the findings of previous studies about the FAM, which also demonstrated correlations between the FAM components.^15,31,51^ The strengths of the correlations were comparable to the mixed (in terms of TBI severity) sample we studied before,^29^ and even higher than the correlations found in the small subgroup of people with mTBI who were studied in a retrospective design.^15^ These findings further confirm the applicability of the FEM to the persistence of symptoms after mTBI. Additionally, as expected in the vicious cycle of the FAM, the results further show a significant and positive correlation between the component depression of the FAM at 3 months post-mTBI and PCS at 6 months post-mTBI.

Unexpectedly, no significant correlations were found between disuse and the other components of the FAM. To the best of our knowledge, the only study in which the relationship between disuse and fear-avoidance behavior and PCS after TBI has been investigated, is the study of Wijenberg.^29^ In contrast with our study, this study included patients with TBI with different severities, from mild to severe TBI. The patients with moderate-to-severe TBI are well known for their negative consequences on the level and frequency of participation (i.e., disuse) in general,^52^ this could potentially explain why Wijenberg^29^ found a significant effect of disuse. Furthermore, the relationship between disuse and PCS in the study of Wijenberg^20^ was very weak and was the weakest correlation within the FAM, which is consistent with our findings.

In a study of the FAM in a (non-TBI) community sample and in multiple chronic pain studies, disuse was the only non-significant factor in the model, which is in line with our results.^20,53,54^ In chronic pain, researchers see that disuse is only seen in high-intensity activities (such as sports), but not in regular daily (non-intensive) activities. In the community sample, the same question was used to measure disuse, which may not be a valid tool. Unfortunately, there are no validated and reliable measurement tools to register disuse in the mTBI population. The instruments used to measure disuse in chronic pain^55^ are unsuitable for the mTBI population because they solely focus on physical activities, whereas in mTBI, mental activities should also be taken into account.

The highest correlation in our study is found between PCS and catastrophizing thoughts at 3 months post-mTBI. Since the FAM represents a disease process that starts with catastrophizing about the PCS, the strong correlation between PCS and catastrophizing at 3 months is meaningful for understanding the mechanism of the FAM in mTBI.^28^ Furthermore, the strong correlation between depressive symptoms at 3 months post-mTBI and PCS at 6 months post-mTBI suggests the existence of the vicious cycle in a longitudinal analysis.

### Strengths and limitations

Our study has several strengths. Most importantly, the study population differs from Wijenberg^15^ and Silverberg^31^ in two ways. Firstly, these studies only included patients in clinical care who were stuck in their recovery. Secondly, these studies included mixed groups in terms of injury severity (moderate-to-severe injury spectrum). In the current study, we included patients with mTBI at the emergency and neurology department in the acute phase. As a consequence our population is a representative reflection of the mTBI population that reaches the hospital, covering the possible broad range of mTBI recovery trajectories. Furthermore, our sample is a representative reflection of the general mTBI population, since the prevalence of PCS in our study (33%) is in line with recent random-effects meta-analyses that showed a PCS prevalence of 31.3% at 3–6 months post-injury.^56^ Another strength is that, contrary to previous studies that did not measure fixed time points post-mTBI, all participants were measured at 3 months post-mTBI. This is of importance as symptoms after mTBI are classified as persistent from 3 months post-mTBI onward.^1–3^ A third strength is that we included a longitudinal perspective on the vicious cycle of the FAM by including the level of the PCS at 6 months post-injury.

There are also limitations to consider in the current study. It is inherent to correlational studies that no conclusions can be drawn regarding mediation or causation. However, we did add a longitudinal element by incorporating two time points. Furthermore, there is no validated method to measure disuse available for the mTBI population. The FMA and PCS-CS questionnaires have already been used in previous research^15^ and a recent study showed adequate psychometric qualities,^42^ but further validation of these questionnaires is necessary for the mTBI population. Additionally, the method of determining PCS through the RPQ score is contentious. While this method is common in scientific research,^37–41^ it is not utilized systematically in clinical practice to classify PCS. This may reduce the comparability of samples and clinical populations. Finally, we focused on the avoidance of mental activities while people may also avoid physical activities, which may lead to (exacerbation of) symptoms; such as headaches due to sports activities.

### Clinical implications and future research

The correlations we found between the components of the FAM are significant and meaningful for clinical practice. Cassetta and colleagues^57^ showed that high levels of fear avoidance are associated with greater perceived disability in daily life. Treatment programs aimed at catastrophizing and fear-avoidance behavior may be beneficial to people with persistent symptoms post-mTBI. Exposure therapy (a form of cognitive behavioral therapy) is a well-known therapy and is widely used as an effective treatment to reduce catastrophizing and fear-avoidance behavior. Future research should investigate whether exposure therapy is a suitable treatment for persistent symptoms following mTBI. Our research group is currently investigating this in several single-case experimental design (SCED) studies. A first application of intensive exposure therapy showed it is a feasible and efficacious therapy for PCS.^58^ Furthermore, Silverberg^59^ published positive results of graded exposure therapy for persistent PCS after mTBI.

The importance of this study for research on mTBI and PCS is that it suggests that on a group level fear-avoidance behavior is related to persistent PCS. It is very unlikely that avoidance behavior is the only pathway that leads to persistent PCS, given the heterogeneity of the patients with persistent PCS. Endurance behavior is another coping style that may lead to persistent PCS and this needs a different treatment approach.^16,59^ At this point, longitudinal studies including the components of the FAM are needed to further understand the development of persistent PCS over time.

Future research should also collect normative data to establish correct cut-off scores for catastrophizing thoughts and fear-avoidance behavior regarding symptoms after mTBI. This is needed to ensure that these measures reflect the patient’s capabilities and symptoms and allow for accurate score interpretation. Furthermore, future studies should be focused on developing a psychometrically sound measure for disuse.

## Conclusion

The results of our study confirm earlier findings that the FAM could be an explanatory model for the development of persistent PCS. This implies that treatment development for patients with persistent PCS could be aimed at the components of the FAM, such as exposure therapy to reduce avoidance behavior.

## Abbreviations Used

CT: computerized tomopgraphy
EFNS: European Federation of Neurological Societies
FAM: Fear Avoidance Model
FMA: Fear of Mental Activity scale
GCS: Glasgow Coma score
HADS: Hospital Anxiety and Depression Scale
LOC: loss of consciousness
MRI: magnetic resonance imaging
mTBI: mild traumatic brain injury
PCS-CS: Post-Concussion Symptoms Catastrophizing Scale
PTA: post traumatic amnesia
RPQ: Rivermead Post-Concussion Symptoms Questionnaire
SCED: single case experimental design
TBI: traumatic brain injury
WHO: World Health Organization
